# Current knowledge and potential intervention of hexosamine biosynthesis pathway in lung cancer

**DOI:** 10.1186/s12957-023-03226-z

**Published:** 2023-10-26

**Authors:** Yi Zou, Zongkai Liu, Wenjia Liu, Zhaidong Liu

**Affiliations:** 1 College of First Clinical Medicine, Shandong University of Traditional Chinese Medicine, Jinan, 250000 Shandong China; 2 Department of Oncology, Affiliated Hospital of Shandong University of Traditional Chinese Medicine, Jinan, 250000 Shandong China

**Keywords:** Lung cancer, Metabolism, Hexosamine biosynthesis pathway, Intervention

## Abstract

Lung cancer is a highly prevalent malignancy characterized by significant metabolic alterations. Understanding the metabolic rewiring in lung cancer is crucial for the development of effective therapeutic strategies. The hexosamine biosynthesis pathway (HBP) is a metabolic pathway that plays a vital role in cellular metabolism and has been implicated in various cancers, including lung cancer. Abnormal activation of HBP is involved in the proliferation, progression, metastasis, and drug resistance of tumor cells. In this review, we will discuss the function and regulation of metabolic enzymes related to HBP in lung cancer. Furthermore, the implications of targeting the HBP for lung cancer treatment are also discussed, along with the challenges and future directions in this field. This review provides a comprehensive understanding of the role and intervention of HBP in lung cancer. Future research focusing on the HBP in lung cancer is essential to uncover novel treatment strategies and improve patient outcomes.

## Introduction

Lung cancer, characterized by a high mortality rate, is among the most frequently diagnosed cancers globally. It is responsible for a significant number of cancer-related deaths each year, as indicated by global statistics [1]. There are two primary types of lung cancer: non-small cell lung cancer (NSCLC) and small cell lung cancer (SCLC). NSCLC, accounting for approximately 80–85% of cases, is the most prevalent form, while SCLC represents about 10–15% of cases [2]. Despite the advancements in diagnosis and treatment, lung cancer continues to exhibit elevated morbidity and mortality rates.

Studies have demonstrated that dysregulated metabolism equips cancer cells with the necessary energy and building blocks to sustain uncontrolled growth and proliferation [3]. Metabolic changes not only fuel tumor cell proliferation but also contribute to other malignant characteristics, including resistance to cell death, immune evasion, and metastasis [3]. Additionally, cancer cells reconfigure their metabolism to support biosynthesis, generating macromolecules essential for cell division and growth. Hence, comprehending the intricate relationship between cell metabolism and tumor development is critical for identifying potential therapeutic targets and developing effective strategies to combat cancer. Targeting metabolic vulnerabilities in cancer cells has emerged as a promising therapeutic strategy [4]. (a) One such vulnerability is the enhanced dependence of cancer cells on glucose metabolism, known as the Warburg effect. Preclinical studies have demonstrated the efficacy of inhibitors targeting glycolysis, such as 2-deoxyglucose (2-DG), in impeding glucose uptake and metabolism, leading to impaired energy production and reduced tumor growth [5]. (b) Another metabolic vulnerability is cancer cells’ reliance on glutamine metabolism. Glutaminase inhibitors, like CB-839, have displayed efficacy in various cancer models, including lung cancer, by depriving cancer cells of glutamine, an indispensable nutrient for biosynthesis and energy production [6]. (c) Additionally, targeting fatty acid metabolism has garnered attention as a potential therapeutic approach. Inhibitors of fatty acid synthase (FASN), an enzyme involved in de novo fatty acid synthesis, have exhibited anti-tumor effects and enhanced sensitivity to chemotherapy in preclinical studies [7]. Overall, targeting metabolic vulnerabilities in cancer cells represents a promising avenue for the development of novel and effective therapeutic strategies.

**Fig. 1:**
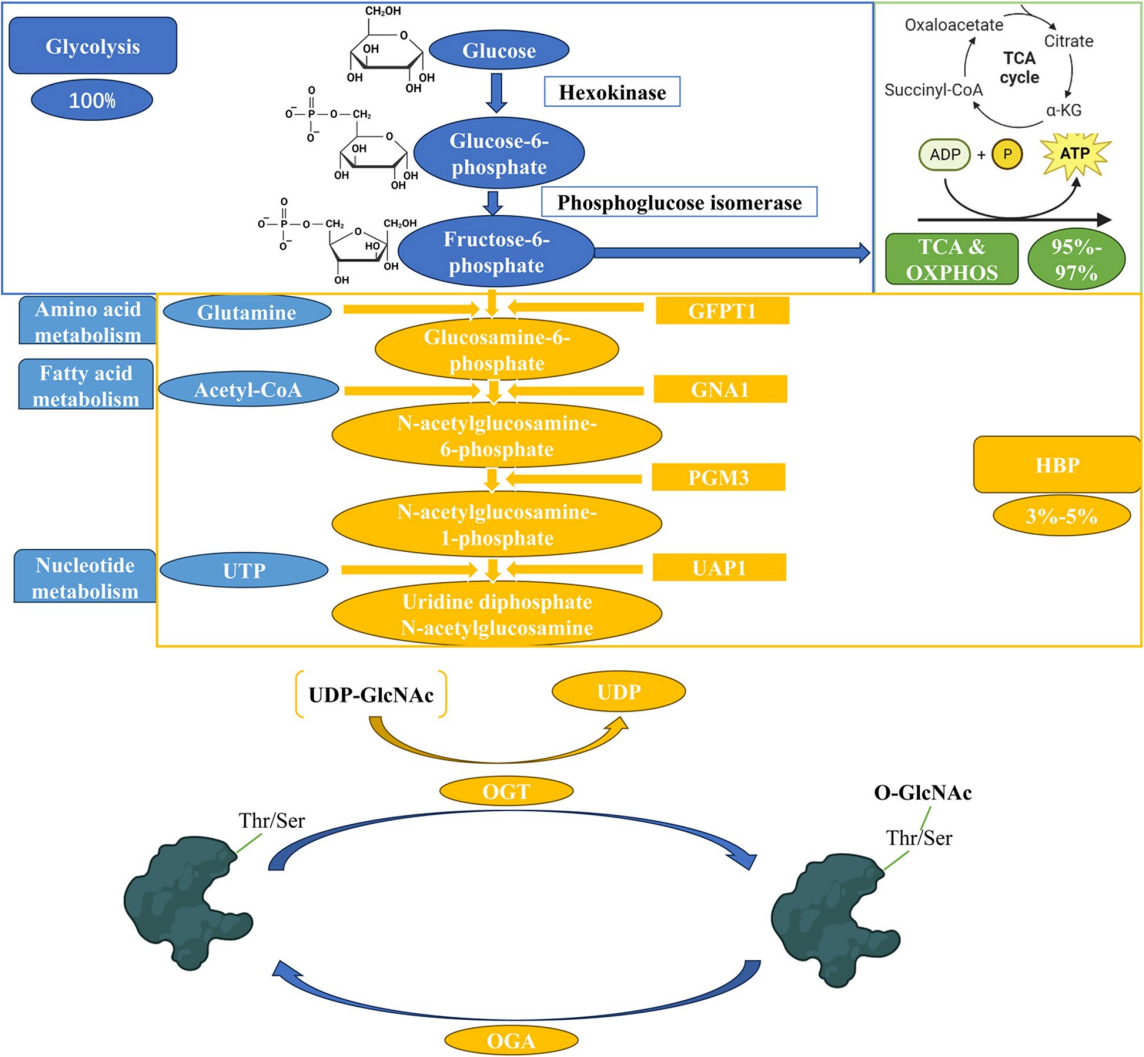
Schematic illustration of Hexosamine biosynthetic pathway and protein O-GlcNAcylation. Glucose-6-phosphate isomerase converts glucose to fructose-6-phosphate. Both fructose-6-phosphate and glucose-6-phosphate can be metabolized through glycolysis or the hexosamine biosynthetic pathway (HBP). The rate-limiting enzyme of the HBP, known as GFPT1 or GFAT, catalyzes the conversion of fructose-6-phosphate to glucosamine-6-phosphate using glutamine as the amide donor. The expression of GFAT mRNA and protein is upregulated by saturated fatty acids, while feedback inhibition occurs due to its enzymatic product, glucosamine-6-phosphate. Subsequently, the enzyme GNA1 converts acetyl-CoA and glucosamine-6-phosphate to CoA and N-acetylglucosamine-6-phosphate. Another enzyme, PGM3, converts N-acetylglucosamine-6-phosphate to N-acetylglucosamine-1-phosphate, with glucose-1,6-bisphosphate serving as a co-factor. Finally, UDP-N-acetylglucosamine is synthesized by adding UTP to N-acetylglucosamine through the action of UAP1

The hexosamine biosynthesis pathway (HBP) is a critical metabolic pathway involved in the synthesis of nucleotide sugars and protein glycosylation [8] (Fig. 1). Nucleotide sugars, generated by the HBP, play an indispensable role as fundamental building blocks in the glycosylation process of proteins, lipids, and other cellular components. Protein O-glycosylation or N-glycosylation, a tightly regulated post-translational modification, exerts a significant influence on protein structure, stability, and function [9]. In the context of cancer, dysregulation of protein O-glycosylation has been implicated in various aspects of tumor progression and metastasis [10]. Anomalous O-glycosylation patterns observed on cancer-associated proteins can enhance tumor cell migration, invasion, and angiogenesis, thereby contributing to metastatic dissemination [11]. It is well known that the O-glycosylation could cause the forming of Tn antigen which is highly expressed in many cancers and associated with high metastatic potential and poor prognosis [11]. Furthermore, altered glycosylation has the capacity to modulate immune recognition and response, thereby facilitating immune evasion by cancer cells [10]. A comprehensive understanding of the significance of HBP in cancer biology can provide valuable insights into the potential of HBP-based interventions to enhance the clinical management and outcomes of patients with lung cancer.

### Role of HBP in cancer

#### Function of HBP

The HBP is a crucial metabolic pathway involved in cellular processes through the regulation of nucleotide sugar synthesis and protein glycosylation. Key enzymes in the HBP, including glutamine fructose-6-phosphate amidotransferase 1 (GFAT1), catalyze the conversion of fructose-6-phosphate and glutamine into glucosamine-6-phosphate and subsequently UDP-GlcNAc [12]. UDP-GlcNAc serves as a critical substrate for protein glycosylation, a post-translational modification involving the attachment of sugar moieties to proteins [13]. Protein glycosylation plays diverse roles in cellular functions such as protein folding, stability, trafficking, and cell–cell interactions [9]. Aberrant protein glycosylation has been implicated in tumor growth, invasion, and immune escape mechanisms, emphasizing the significance of the HBP in cancer biology [14].

The HBP is tightly regulated to ensure proper functioning and integration with cellular metabolism. Glucose availability is a key regulator of the HBP since glucose is the primary substrate for the pathway, directly influencing flux [15]. Increased glucose uptake and metabolism in cancer cells have been shown to enhance HBP activity and UDP-GlcNAc production [16], indicating a close relationship between glucose metabolism and HBP regulation in cancer cells. Additionally, several signaling pathways, including the mTOR (mechanistic target of rapamycin kinase) pathway, a central regulator of cell growth and metabolism, have been implicated in HBP regulation [17]. Activation of mTOR signaling promotes HBP flux and increases UDP-GlcNAc levels. Furthermore, the O-GlcNAc transferase (OGT), responsible for O-GlcNAcylation, which adds GlcNAc moieties to nuclear and cytoplasmic proteins [18], has been linked to HBP regulation. OGT can sense nutrient availability and modulate HBP enzyme expression [19].

#### HBP and tumorigenesis

Activation of the HBP promotes cancer cell proliferation by providing necessary substrates for nucleotide sugar synthesis and protein glycosylation, both involved in cell growth and signaling pathways [16]. Increased HBP activity is associated with enhanced cancer cell survival and resistance to apoptosis, potentially through the modulation of cellular stress responses and anti-apoptotic pathways [20]. Moreover, the HBP has been implicated in cancer cell metastasis, a major determinant of cancer progression and poor patient prognosis [21]. Studies have demonstrated that HBP activation promotes epithelial-mesenchymal transition (EMT), a process associated with increased invasiveness and metastatic potential of cancer cells [22]. HBP-mediated protein glycosylation regulates cell adhesion molecules, extracellular matrix remodeling, and angiogenesis, all contributing to the metastatic cascade [10]. In the context of tumor immunity, the HBP has emerged as a regulator of immune cell function and tumor immune evasion. Dysregulation of the HBP in cancer cells can lead to aberrant protein O-glycosylation patterns, affecting immune cell recognition and interaction with tumor cells [23]. Altered glycosylation impacts immune cell signaling, cytokine production, and antigen presentation, ultimately influencing the antitumor immune response [24].

#### HBP and O-GlcNAcylation

Dysregulation of protein glycosylation by the HBP can impact cell adhesion, signaling, and immune response. Altered glycosylation patterns have been observed in various cancers and are associated with tumor growth, invasion, and metastasis [25, 26]. For instance, a study by Zhu et al. revealed that N-glycosylation of CD82, a member of the tetraspanin superfamily, at the Asn157 site inhibits EMT by down-regulating the Wnt (wingless)/β-catenin pathway, resulting in reduced lung metastases of colorectal cancer [25]. The HBP also modulates signaling pathways involved in cancer progression. Notably, the O-GlcNAcylation of c-MYC by the OGT enzyme at Thr58 is essential for c-MYC stability [27]. HBP-mediated protein O-GlcNAcylation plays a regulatory role in the activity of proteins associated with cell cycle progression, apoptosis, and oncogenic signaling pathways, such as the Wnt/β-catenin and NF-κB (Nuclear factor kappa B) pathways. Specifically, the knockdown of GFAT1, which is the rate-limiting enzyme of the HBP, leads to the inhibition of β-catenin activity and the transcription of downstream target genes *CCND1* (Cyclin D1) and *MYC* [28]. Importantly, Taparra et al. conducted a significant study revealing the essential role of HBP in inhibiting KRAS (KRAS proto-oncogene) G12D-induced senescence and its impact on lung tumorigenesis. The study demonstrated that suppressing HBP significantly attenuates KRASG12D-induced lung tumorigenesis. Mechanistically, the O-GlcNAcylation of SNAI1 and c-MYC was found to be correlated with the EMT-HBP pathway, thereby accelerating lung tumorigenesis [29].

#### HBP and other metabolic pathways

The interplay between the HBP and other metabolic pathways is essential for cancer cell metabolism and progression. A critical interaction occurs between the HBP and the highly active glycolytic pathway in cancer cells. Recent findings by Maucieri et al. revealed that suppression of O-GlcNAcylation in Granulosa cells impaired the expression of hexokinase and pyruvate kinase [30]. Furthermore, HBP-mediated protein O-GlcNAcylation can influence the stability and function of glycolytic enzymes, thereby impacting their enzymatic activity. Nie et al. demonstrated elevated levels of O-GlcNAcylation on phosphoglycerate kinase 1 (PGK1), the initial ATP (adenosinetriphosphate)-producing enzyme in glycolysis, at threonine 255 in human colon cancers [31]. Notably, O-GlcNAcylation was found to enhance PGK1 enzymatic activity, promoting lactate production, while simultaneously causing the translocation of PGK1 into the mitochondria to inhibit the pyruvate dehydrogenase (PDH) complex and downregulate oxidative phosphorylation. These results suggest that O-GlcNAcylation plays a role in coordinating glycolysis and the TCA (tricarboxylic acid) cycle, thereby promoting colon cancer tumorigenesis. Furthermore, Munemoto et al. demonstrated the activation of the HBP pathway and upregulation of OGT expression in esophageal cancer [32]. Interestingly, they also observed a greater activation of the PPP in higher degrees of esophageal cancer malignancy. However, further investigation is needed to determine the involvement of the HBP in regulating PPP during tumorigenesis and development.

### Regulation of HBP in lung cancer

#### GFAT1

**Fig. 2:**
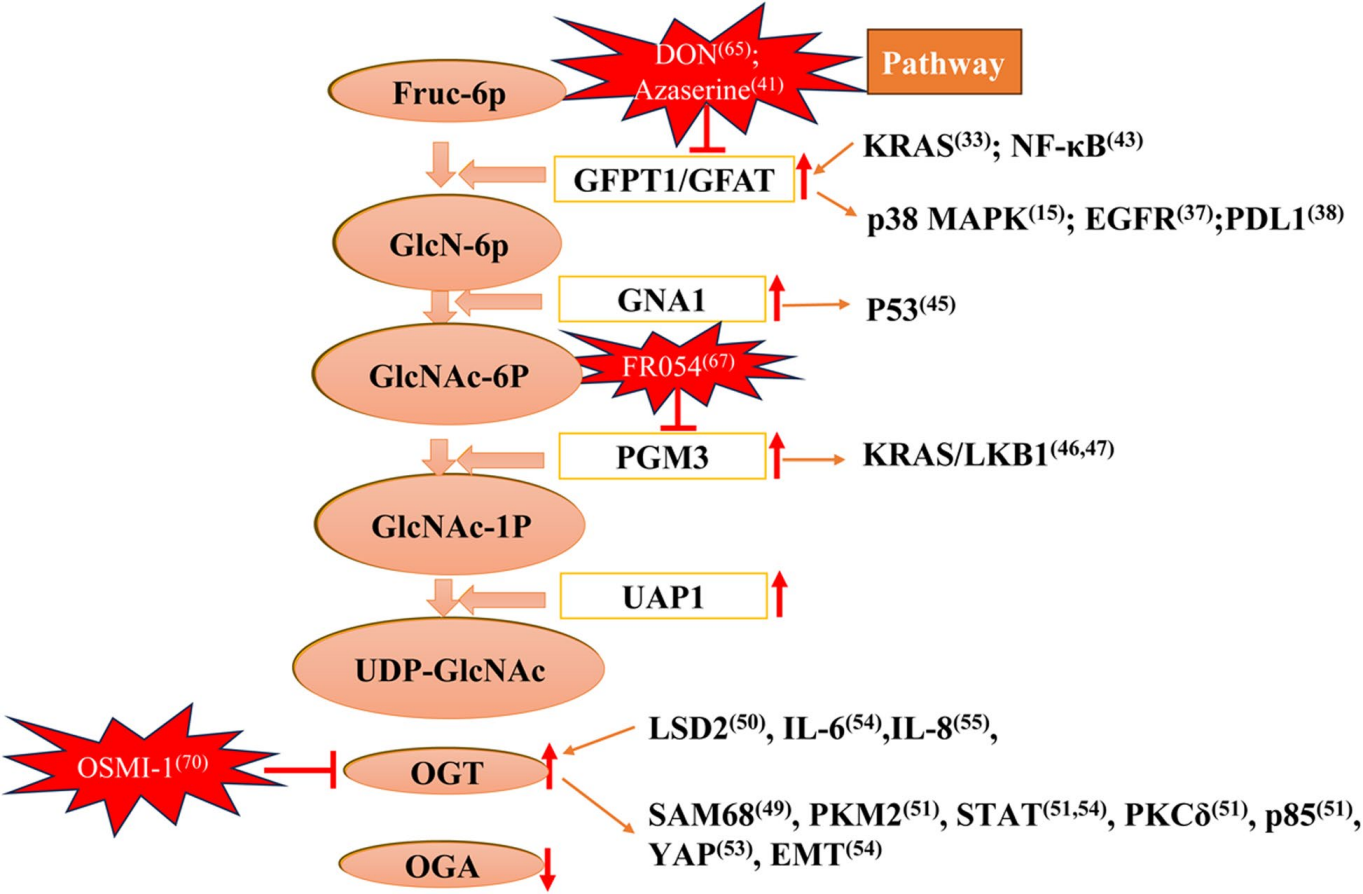
The molecular mechanism of HBP involved in lung cancer. Several small molecular compounds (DON, Azaserinethat, FR054, OSMI-1) target HBP enzymes are illustrated. Glc-6P, glucose-6-phosphate; Fruc-6P, fructose-6-phosphate; GPI, glucose-6-phosphate isomerase; GFPT1 or GFAT, glutamine-fructose 6-phosphate aminotransferase; GlcN-6P, glucosamine-6-phosphate; GNA1, d-glucosamine-6-phosphate N-Acetyltransferase; GlcNAc-6P, N-acetylglucosamine-6-phosphate; GlcNAc-1P, N-acetyl-1-phosphate glucosamine

The involvement of the HBP in the development and progression of lung cancer has been extensively studied, providing insights into the molecular mechanisms underlying its role in lung cancer pathogenesis (Fig. 2 and Table 1). Recently, Wei et al. reported a correlation between the expression of GFAT1, the rate-limiting enzyme of the HBP, and poor prognosis in patients with lung adenocarcinoma (LUAD) [12]. Mechanistically, GFAT1 was found to facilitate the formation of a TTLL5 (tubulin tyrosine ligase like 5)-GFAT1-TAB1 (TGF-beta activated kinase 1-binding protein 1) complex by binding to TAB1. This complex formation, coupled with GFAT1’s activity in glutamate production, contributed to TTLL5-mediated TAB1 glutamylation, promoting p38 MAPK activation and LUAD malignancy. Another study by Dragic et al. revealed the critical role of the HBP in LUAD cell survival under glucose shortage conditions [33]. They demonstrated that the HBP rescued the cell surface expression of specific glycoproteins, including epidermal growth factor receptor (EGFR), in the glucose-deficient tumor microenvironment. Notably, a correlation between GFAT1 and wild-type EGFR activation in LUAD was uncovered [33]. Furthermore, the stability of programmed death-ligand 1 (PD-L1) protein was found to depend on its glycosylation, with GFAT1 playing a crucial role in maintaining PD-L1 stability [34]. Inhibition of GFAT1 activity significantly reduced IFNγ-induced PD-L1 levels in various lung cancer cell lines by suppressing PD-L1 glycosylation and promoting its proteasomal degradation [34]. These findings suggest the potential use of GFAT1 inhibitors to modulate PD-L1 protein levels, thereby enhancing the efficacy of immunotherapy for lung cancer.

**Table 1:** Regulation of HBP in lung cancer

Genes	Targets/pathways	Function	In vitro/in vivo	Reference
GFAT1	TAB1/MAPK	Autophagy↑cell survival↑	In vitro and in vivo	[12]
	EGFR	Cell survival↑	In vitro	[33]
	PD-L1	Tumor immunity↓	In vitro	[34]
GFPT1/2	KRAS	Senescence↑cell growth↑	In vitro and in vivo	[29, 35]
	SIRT6/NF-κB	Cell migration and invasion↑	In vitro	[36]
GNPNAT1	p53	Immune infiltration↓	In vitro	[37]
PGM3	KRAS/LKB1	Cell proliferation↑	In vitro and in vivo	[38]
UAP1	…	Cell proliferation↑	In vitro	[39]
OGT	SAM68	Cell migration and invasion↑	In vitro	[40]
	LSD2	Cell proliferation↑	In vitro	[41]
	PKM2	Cell proliferation and differentiation↑	In vitro	[42]
	STAT3	Migration and invasion↑	In vitro	[43]
OGA	…	…	…	…

*Note*: “↑”: promote; “↓”: inhibit; “…”: not available

#### GFPT1/2

In the initial rate-limiting step of the HBP, the conversion of fructose-6-phosphate to glucosamine-6-phosphate is catalyzed by GFPT1/2 enzymes. This enzymatic process controls the influx of glucose into the HBP [44]. Recent evidence suggests that aberrant KRAS signaling promotes increased glucose flux into the HBP [45]. HBP has been found to be essential for suppressing KRASG12D-induced senescence, and targeting HBP significantly delays lung tumorigenesis driven by KRASG12D [29]. A metabolomic analysis conducted by Kim et al. on genetically engineered mouse models combining oncogenic KRAS with LKB1 mutations revealed the activation of the HBP pathway in NSCLC [35]. Additionally, they demonstrated that selectively inhibiting the HBP enzyme GFPT2 led to reduced growth of KRAS/LKB1 co-mutant tumor cells in culture, xenografts, and genetically modified mice. Furthermore, Zhang et al. reported the critical role of GFPT2 in regulating tumor metabolic reprogramming in LUAD, suggesting that the HBP could serve as a potential therapeutic target for LUAD treatment [36]. Analyzing transcriptomic data, Szymura et al. identified *GFPT2* as one of the most significantly upregulated metabolic genes in mesenchymal NSCLC [46]. They also demonstrated that *GFPT2* is transcriptionally upregulated by NF-κB and repressed by the NAD^+^-dependent deacetylase SIRT (Sirtuin) 6. Depleting GFPT2 in NSCLC resulted in attenuated cell migration and invasion, indicating the potential importance of modulating GFPT2 activity as a therapeutic target for combating NSCLC progression.

#### GNPNAT1

In the second step of the HBP, the conversion of glucosamine-6-phosphate to N-acetylglucosamine-6-phosphate is catalyzed by glucosamine 6-phosphate N-acetyltransferase (GNPNAT1) [47]. Although limited, reports have implicated GNPNAT1 in lung cancer. Recent findings have shown that GNPNAT1, a key enzyme in the HBP, is upregulated in LUAD compared to normal tissues and is associated with clinical stage, tumor size, and lymphatic metastasis status in patients [37]. Kaplan–Meier (KM) analysis further revealed that increased GNPNAT1 expression is correlated with a relatively poor prognosis. Multivariate Cox regression analysis confirmed GNPNAT1 as an independent prognostic factor for LUAD. Additionally, gene set enrichment analysis (GSEA) indicated a link between GNPNAT1 and signaling pathways involved in ubiquitin-mediated proteolysis and p53.

#### PGM3

N-acetylglucosamine-phosphate mutase 3 (PGM3) plays a pivotal role in the HBP by converting N-acetylglucosamine-6-phosphate to N-acetylglucosamine-1-phosphate. However, there is limited research on the involvement of PGM3 in lung cancer. In a study by Lee et al., they discovered that KRAS/LKB1 co-mutant cells exhibit an increased dependency on PGM3, an enzyme downstream of GFPT2 [38]. Through genetic or pharmacological suppression of *PGM3*, they observed a reduction in the growth of KRAS/LKB1 co-mutant tumors both in vitro and in vivo. These findings identify a metabolic vulnerability specific to KRAS/LKB1 co-mutant tumors within the HBP and propose the targeting of PGM3 as a potential therapeutic strategy for this aggressive subtype of NSCLC. Additionally, it has been reported that cancer cells harboring oncogenic KRAS rely heavily on HBP for their growth and survival [48]. Seo et al. developed a quantitative method using ultra-high-performance liquid chromatography-tandem mass spectrometry to distinguish between HBP metabolites in KRAS wild-type and mutant lung cancer cells, providing a valuable tool for metabolic research in cancer and the development of new anticancer drugs [48].

#### UAP1

UAP1, the final enzyme and second rate-limiting step in the HBP, is responsible for generating UDP-GlcNAc, a high-energy sugar donor crucial for subsequent glycosylation, particularly O-GlcNAcylation of intracellular target proteins. Through bioinformatics analysis, Wang et al. revealed that UAP1 is upregulated in LUAD tissues and is associated with poor clinical outcomes [39]. Furthermore, high levels of *UAP1* expression correlated with larger tumor sizes and advanced TNM stages in LUAD patients.

#### OGT

OGT, an essential HBP enzyme, facilitates the transfer of a GlcNAc residue from UDP-GlcNAc to serine or threonine residues on intracellular target proteins. In a study by Lin et al., they conducted an O-GlcNAc proteome profiling of LUAD cell lines and identified SAM68 as a nuclear protein undergoing O-GlcNAcylation and potential interaction with OGT [40]. Importantly, they discovered that multiple serine residues in SAM68’s N-terminal region play a critical role in its O-GlcNAcylation and its regulatory function in lung cancer cell migration and invasion. These findings emphasize the significance of O-GlcNAcylation on SAM68 (KH RNA-binding domain containing, signal transduction associated 1) in driving the aggressiveness of LUAD. Additionally, Yang et al. reported that LSD2 (lysine demethylase 1B), a well-known histone H3K4me1/me2 demethylase, directly ubiquitylates and promotes proteasome-dependent degradation of OGT, resulting in the inhibition of lung cancer cell growth [41]. This study highlights the pivotal role of LSD2 in suppressing lung cancer growth through the regulation of OGT. Another study by Yang et al. revealed that EGF stimulation enhances OGT binding to PKM2 by promoting OGT Y976 phosphorylation [42]. Consequently, this leads to elevated PKM2 O-GlcNAcylation and tetramerization, ultimately causing a significant decrease in PKM2 activity. Notably, they also observed an enhanced association between OGT and additional phosphotyrosine-binding proteins such as STAT (signal transducer and activator of transcription)1, STAT3, STAT5, PKCδ, and p85 when Y976 was phosphorylated [42].

It has been reported that Yes-associated protein (YAP), a proto-oncoprotein, undergoes inhibition following phosphorylation by the tumor-suppressing Hippo pathway [49]. Recently, it has been discovered that O-GlcNAcylation suppresses YAP phosphorylation in LUAD [50]. Ge et al. reported that IL-6 stimulation increases OGT expression and overall protein O-GlcNacylation in A549 cells [43]. Additionally, they found that silencing OGT significantly inhibits IL-6-induced expression of EMT markers (N-cadherin and Slug), as well as migration and invasion in A549 cells. Mechanistically, OGT interacts with STAT3 and mediates its O-GlcNacylation, thereby promoting STAT3 Y705 phosphorylation in IL-6-treated NSCLC cells. Shimizu et al. also demonstrated that IL-8 stimulation of lung cancer cells enhances glucose uptake and the expression of glucose transporter 3 (GLUT3) and glucosamine fructose-6-phosphate aminotransferase (GFAT), a regulator of glucose flux in the HBP, resulting in increased protein O-GlcNAcylation [51]. Importantly, they showed that OSMI1, an O-GlcNAcylation inhibitor, reduces the number of cancer stem cells (CSCs) and tumor development in vivo. Furthermore, numerous studies have established a correlation between aberrant O-GlcNAcylation and cisplatin resistance in lung cancer [52, 53]. Luanpitpong et al. have reported the implication of O-GlcNAcylation of p53/c-Myc in cisplatin-induced apoptosis [53]. Wang et al. have similarly observed that cisplatin induces protein O‑GlcNAcylation by modulating the activity of OGT, OGA, and AMPK in lung cancer [52].

#### OGA

In contrast to OGT, O-GlcNAcase (OGA) is responsible for removing GlcNAc residues from target proteins. Previous work by Hwang et al. reported a downregulation of O-GlcNAcylation levels and an increase in OGA expression in LPS-induced lung tissues of mice [54]. Interestingly, OGA inhibitors have been shown to suppress the inflammatory response to LPS, both in vitro and in vivo, suggesting a role for OGA in inflammation during lung injury. Chatterjee et al. also reported an association between OGA and lung endothelial cell injury [55]. More recently, the involvement of OGA in lung cancer has been investigated. Despite the general decrease in protein O-GlcNAcylation under conditions of glucose deprivation, studies have demonstrated increased O-GlcNAcylation in A549 cells in response to glucose deprivation [56]. Moreover, they revealed that the increased O-GlcNAcylation is attributed to elevated OGT activity and decreased OGA activity, indicating that posttranslational regulation of OGA likely influences its activity under glucose deprivation conditions.

Furthermore, research has explored the therapeutic potential of targeting the HBP in lung cancer. For instance, Chen et al. demonstrated that inhibiting GFAT1 sensitizes lung cancer cells to chemotherapy, suggesting the possibility of combining HBP-targeting agents with conventional therapies to enhance treatment outcomes [52]. Luanpitpong et al. identified hyper-O-GlcNAcylation of p53/c-Myc in lung carcinoma cells through OGA inhibition, resulting in resistance to apoptosis induced by cisplatin [53]. Additionally, Chen et al. reported increased expression of GFAT in NSCLC cell lines and tissues and showed that inhibiting GFAT activity or knocking down GFAT impairs cell proliferation and enhances cytotoxicity when combined with cisplatin treatment [57]. Mechanistically, suppressing GFAT activity downregulates BiP (glucose-regulated protein 78), which activates inositol-requiring enzyme 1α, a sensor protein of the unfolded protein response (UPR), and exacerbates cisplatin-induced apoptosis. These findings highlight the potential of targeting the GFAT-mediated HBP to improve platinum-based chemotherapy for NSCLC [57]. Collectively, these studies provide compelling evidence for the involvement of the HBP in the development and progression of lung cancer. However, further exploration is warranted to fully understand the intricate mechanisms underlying the role of the HBP in lung cancer.

#### Potential intervention strategies targeting HBP in lung cancer

Recent evidence has increasingly highlighted the significance of elevated O-GlcNAcylation levels as a crucial hallmark in several cancer types [58, 59]. Importantly, targeting the HBP as a means of metabolic reprogramming has demonstrated therapeutic potential that is specific to cancer cells, with minimal impact on normal cells’ O-GlcNAcylation status [60]. Thus, the HBP emerges as a promising and innovative target for cancer treatment. Encouragingly, numerous inhibitors have been developed and extensively tested in both cellular and animal models to target HBP enzymes (Fig. 2).

Small molecule inhibitors offer potential therapeutic strategies to modulate the dysregulated HBP in lung cancer, thereby influencing cancer cell behavior and improving treatment outcomes. Notably, inhibition of GFAT1 has shown promising anti-tumor effects by suppressing HBP activity and reducing cancer cell proliferation [12]. Targeting GFAT1 has also demonstrated the ability to sensitize lung cancer cells to chemotherapy and enhance treatment responses [61]. Sharma et al. revealed that DON, a small molecule glutamine analog that targets GFAT1, decreases the self-renewal potential and metastatic abilities of tumor cells [61]. Moreover, DON treatment leads to reduced hyaluronan and collagen levels in the tumor microenvironment, resulting in extensive remodeling of the extracellular matrix (ECM) and increased infiltration of CD8 + T cells. Excitingly, DON treatment has also been shown to sensitize pancreatic tumors to anti-PD1 therapy, leading to tumor regression and prolonged survival [61]. While DON has exhibited promising efficacy in clinical trials, its application is limited by gastrointestinal (GI) toxicity [62]. Therefore, modifying and optimizing the drug’s structure to develop prodrugs with reduced GI toxicity holds significant value. Despite the lack of intervention studies specifically in lung cancer, the disruptive potential of DON on HBP-mediated signaling pathways positions it as a promising candidate for combination therapy in lung cancer treatment.

In recent investigations, inhibitors targeting the PGM3 enzyme have emerged as potential therapeutic agents for various cancer types. Ricciardiello et al. conducted preclinical evaluations of FR054, a novel PGM3 inhibitor, and observed its remarkable anti-breast cancer effects [63]. They found that FR054 induces an early proliferation arrest followed by a substantial increase in cell death in breast cancer cells [63]. Furthermore, FR054 reduces breast cancer cell adhesion, migration, integrin β1 membrane localization, and endoplasmic reticulum (ER) stress. Notably, FR054 also demonstrates reduced tumor growth in breast cancer cell xenograft models. Zerbato et al. reported that FR054 synergistically enhances erastin-induced pancreatic cancer cell death by activating the unfolded protein response [64]. Additionally, Ricciardiello et al. discovered that combined treatment of FR054 with BI-2852, a pan-RAS inhibitor, exerts an additive negative effect on cell proliferation and survival by suppressing Akt activity and cyclin D1 expression. This finding suggests that co-inhibition of the HBP and oncogenic RAS may represent a novel therapeutic strategy for patients with pancreatic ductal adenocarcinoma (PDAC) [65]. Therefore, it is of utmost importance to investigate the inhibitory role of FR054 in the initiation and progression of lung cancer, particularly in the context of KRAS-mutant lung cancer.

OGT, the enzyme responsible for adding O-GlcNAc modifications to target proteins, plays a vital role in regulating the HBP. In lung cancer, inhibiting OGT has been shown to attenuate HBP-mediated glycosylation and disrupt cancer cell growth and survival [52]. OGT inhibitors have emerged as significant inhibitors in various tumor types. Lee et al. reported that treatment with OSMI-1, an OGT inhibitor, synergistically enhanced TRAIL-induced apoptosis signaling in HCT116 human colon cancer cells [66]. Mechanistically, OSMI-1 substantially increased TRAIL-mediated apoptosis by upregulating the expression of the cell surface receptor DR5. Crucially, the combination of OSMI-1 and TRAIL exhibited heightened anticancer activity in HCT116 xenograft models in nude mice [66]. Similarly, Yang et al. demonstrated the effective suppression of HCC progression by OSMI-1 [67]. Thus, targeting OGT holds promise as a therapeutic approach to inhibit HBP-driven oncogenic processes in lung cancer.

In KRAS/LKB1 co-mutant lung cancer cells, Kim et al. reported that azaserine, a GFPT inhibitor, significantly enhanced cell death in vitro [35]. Moreover, azaserine monotherapy demonstrated reduced tumor burden in KRAS/LKB1 co-mutant models. Azaserine presents a promising therapeutic strategy to modulate HBP-related metabolic processes in lung cancer.

The interplay between the HBP and other metabolic pathways, such as glycolysis and the pentose phosphate pathway (PPP), offers additional targets for potential interventions. Modulating key enzymes or regulators involved in metabolic cross-talk, such as phosphofructokinase, pyruvate kinase, and pyruvate dehydrogenase in aerobic glycolysis, could impact HBP activity, influencing lung cancer cell metabolism and survival [30]. This modulation may help restore metabolic homeostasis, inhibit tumor growth, and overcome drug resistance.

Combination therapies involving HBP-targeting agents have shown promise in enhancing the efficacy of cancer treatment. Combining GFAT inhibitors, which target the HBP, with conventional chemotherapeutic drugs has been demonstrated to synergistically inhibit tumor growth in lung cancer treatment. For instance, the combination of a GFAT inhibitor and cisplatin, a commonly used chemotherapy drug, exhibited superior inhibitory effects compared to individual treatments [57]. Additionally, combining HBP inhibition with immune checkpoint inhibitors has been explored as a strategy to enhance the anti-tumor immune response. Studies have also suggested that combining HBP-targeting agents with radiotherapy can improve the radiosensitivity of cancer cells [68]. These findings underscore the potential benefits of combining HBP-targeting agents with other treatment modalities in lung cancer therapy.

To date, despite numerous in vitro and in vivo experiments demonstrating the therapeutic potential of inhibitors directed at the HBP pathway in cancer treatment, these inhibitors have not yet progressed to clinical trials. As a result, it is imperative to assess the clinical efficacy of HBP pathway inhibitors in the context of anti-tumor strategies. Furthermore, additional research and clinical investigations are warranted to refine and optimize these combinatorial approaches.

## Conclusion

The HBP has emerged as a critical player in the metabolic reprogramming of cancer cells, including those in lung cancer. It plays a significant role in nucleotide sugar synthesis and protein glycosylation, processes that contribute to various cellular events crucial for cancer progression, metastasis, and immune evasion [16]. Dysregulation of the HBP has been observed in lung cancer, leading to altered metabolism and signaling pathways that drive tumor growth and survival [37]. This dysregulation is influenced by upstream regulatory mechanisms, including changes in nutrient availability, signaling pathways, and transcription factors [21], resulting in alterations in the expression and activity of HBP enzymes. Dysregulated HBP further impacts downstream signaling pathways and molecular targets, including the PI3K (phosphatidylinositol 3 kinase)/AKT pathway [69], thereby contributing to lung cancer progression. Targeting the dysregulated HBP in lung cancer represents a promising therapeutic strategy. Several approaches, such as small molecule inhibitors targeting HBP enzymes like GFAT1, have shown promising anti-tumor effects in preclinical studies [61]. Additionally, combination therapies involving HBP-targeting agents, such as HBP inhibitors in combination with chemotherapy, targeted therapies, or radiotherapy, have demonstrated synergistic effects in enhancing treatment outcomes.

### Future perspectives

Despite significant progress in understanding the role and therapeutic potential of the HBP in lung cancer, there are still knowledge gaps and avenues for future research. Firstly, a more comprehensive understanding of the precise molecular mechanisms underlying HBP dysregulation and its interplay with other metabolic pathways in lung cancer is needed. This understanding will aid in identifying key targets for therapeutic intervention and developing more precise treatment strategies.

Furthermore, the identification of biomarkers associated with HBP dysregulation and treatment response is crucial for patient stratification and personalized therapies. Biomarkers can provide valuable insights into predicting patient outcomes, monitoring treatment responses, and unraveling mechanisms of resistance. Integrating multi-omics approaches, such as genomics, transcriptomics, proteomics, and metabolomics, will offer a comprehensive understanding of the HBP and its intricate interactions within the tumor microenvironment.

Furthermore, it is essential to explore the potential clinical implications of targeting the HBP in lung cancer. Conducting clinical trials to evaluate the efficacy, safety, and potential for improved patient outcomes of HBP-targeting agents, both as monotherapies and in combination with standard treatments, is a necessary step. Additionally, investigating the role of HBP dysregulation in specific subtypes of lung cancer and its association with clinical parameters, such as tumor stage, metastasis, and patient survival, will contribute to a better understanding of its clinical relevance.

Despite the promising potential of HBP-targeting interventions, several challenges need to be addressed. One challenge involves developing specific and potent inhibitors that selectively target HBP enzymes without affecting normal cellular functions. Furthermore, understanding the potential off-target effects and toxicity profiles of HBP inhibitors is crucial for their successful translation into clinical practice.

Another significant challenge is overcoming drug resistance mechanisms that may arise during HBP-targeted therapies. Long-term efficacy and the emergence of resistance remain important concerns that should be addressed through combination therapies, rational drug design, and the identification of synergistic treatment approaches.

Future investigations should focus on filling the knowledge gaps, exploring novel biomarkers, conducting rigorous clinical trials, and overcoming challenges associated with HBP-targeting interventions. Advancements in these areas will not only enhance our understanding of the clinical implications of the HBP but also pave the way for the development of effective and personalized treatment strategies for patients with lung cancer.

## Data Availability

Data sharing is not applicable to this article as no datasets were generated or analyzed during the current study.

## Abbreviations

HBP: Hexosamine biosynthesis pathway
NSCLC: Non-small cell lung cancer
SCLC: Small cell lung cancer
2-DG: 2-Deoxyglucose
FASN: Fatty acid synthase
GFAT1: Glutamine fructose-6-phosphate amidotransferase 1
mTOR: Mechanistic target of rapamycin kinase
NF-κB: Nuclear factor kappa B
OGT: O-GlcNAc transferase
EMT: Epithelial-mesenchymal transition
CCND1: Cyclin D1
MerTK: Mer tyrosine kinase
HCC: Hepatocellular carcinoma
PGK1: Phosphoglycerate kinase 1
PDH: Pyruvate dehydrogenase
LUAD: Lung adenocarcinoma
EGFR: Epidermal growth factor receptor
PD-L1: Programmed death-ligand 1
GNPNAT1: Glucosamine 6-phosphate N-acetyltransferase
GSEA: Gene set enrichment analysis
TCA: Tricarboxylic acid
TAB1TGF: beta activated kinase 1-binding protein 1
TTLL5: Tubulin tyrosine ligase like 5
PGM3: N-acetylglucosamine-phosphate mutase 3
LSD2: Lysine demethylase 1B
YAP: Yes-associated protein
GLUT3: Glucose transporter 3
SIRT: Sirtuin
SAM68: KH RNA-binding domain containing, signal transduction associated 1
CSCs: Cancer stem cells
OGA: O-GlcNAcase
UPR: Unfolded protein response
STAT: Signal transducer and activator of transcription
ECM: Extracellular matrix
ER: Endoplasmic reticulum
PPP: Pentose phosphate pathway
PI3K: Phosphatidylinositol 3 kinase
